# Association of circadian syndrome with the risk of physical, psychological, and cognitive multimorbidities: a prospective cohort study based on the China Health and Retirement Longitudinal Study

**DOI:** 10.7189/jogh.15.04351

**Published:** 2025-12-19

**Authors:** Jingru Bi, Yilin Pan, Wenkai Guo, Pengcheng Ji, Zenghui Xing, Long Feng, Yuansheng Xie

**Affiliations:** 1 Department of Nephrology, First Medical Center of Chinese PLA General Hospital, State Key Laboratory of Kidney Diseases, National Clinical Research Center for Kidney Diseases, Beijing, China; 2 Nankai University, School of Medicine, Tianjin, China; 3 Beijing Anzhen Hospital, Capital Medical University, Department of Critical Care Medicine, Beijing, China

## Abstract

**Background:**

Multimorbidity involving physical, psychological, and cognitive decline is a major public health challenge with poorly understood upstream risk factors. Circadian syndrome (CircS), which integrates metabolic, sleep, and mood dysregulation, is a potential predictor of this condition. We aimed to investigate the prospective association between baseline CircS and the incidence of distinct multimorbidity patterns.

**Methods:**

We conducted a prospective cohort study of 8262 participants aged ≥45 years from the China Health and Retirement Longitudinal Study, who were free of specified multimorbidity at baseline in 2011. We defined CircS as the presence of ≥4 of 7 components: central obesity, hypertension, dysglycaemia, dyslipidaemia, low high-density lipoprotein cholesterol, abnormal sleep duration, and depressive symptoms. We used multivariable Cox proportional hazards models to estimate hazard ratios (HR) for four incident multimorbidity patterns over a seven-year follow-up.

**Results:**

Over a median follow-up of seven years, baseline CircS was significantly associated with a higher risk of incident physical-psychological-cognitive multimorbidity (HR = 1.48, 95% confidence interval (CI) = 1.09, 2.02) and psychological-cognitive multimorbidity (HR = 1.38; 95% = CI 1.06, 1.79) after full adjustment. We noted a significant dose-response relationship. The population attributable fraction of CircS for physical-psychological-cognitive multimorbidity was 16.8%. Associations were more pronounced in women and participants without baseline chronic conditions.

**Conclusions:**

CircS is a significant, integrative risk factor that precedes the onset of complex multimorbidity, particularly patterns involving cognitive and psychological decline. The co-occurrence of metabolic, sleep, and mood dysregulation appears to synergistically accelerate disease clustering. Our findings identify CircS as a critical target for early risk stratification and suggest that prevention strategies should promote circadian health.

With global ageing and modern lifestyle shifts, multimorbidity, understood as the presence of two or more chronic diseases, has become a major 21st-century public health challenge [1,2]. This is also true for China, where it affected 55.12% adults aged ≥45 years in 2015 [3]. Rather than a random accumulation of diseases, multimorbidity often manifests in specific clusters. Among these, physical, psychological, and cognitive multimorbidity (PPC-MM) is especially frequent in older adults, affecting between 8.1% to 33.9% of this age group globally [4], severely impairing their quality of life and placing strain on healthcare systems. Identifying upstream risk factors for PPC-MM is therefore both a clinical and public health priority.

The circadian system regulates key physiological functions. Governed by the suprachiasmatic nucleus, this system maintains a ~24-hour rhythm *via* core clock genes, which can be disrupted by modern lifestyles, including shift work and nighttime light exposure. The clinical manifestation of this disruption is known as the circadian syndrome (CircS), which encompasses conditions such as central obesity, hypertension, dysregulated glucose and lipid metabolism, sleep disorders, and depressive symptoms. Unlike traditional metabolic syndrome, CircS incorporates sleep and mood disturbances [5,6]. Emerging evidence suggests it to be a potent predictor for cardiovascular and chronic kidney disease [7,8].

Given that individual CircS components (*e.g.* metabolic disorders, sleep disruption, depression) are established risk factors for physical, cognitive, and psychological diseases, we hypothesised that CircS, as an integrated syndrome, could be a critical upstream driver of complex multimorbidity patterns like PPC-MM. However, no prospective studies have investigated the longitudinal association between CircS and the subsequent risk of developing PPC-MM or other multimorbidity patterns.

By utilising data from the China Health and Retirement Longitudinal Study (CHARLS), we aimed to investigate the association of CircS and its component count with the incidence of different multimorbidity patterns, particularly PPC-MM, in a large sample of Chinese middle-aged and older adults.

## METHODS

The CHARLS is a nationally representative cohort of Chinese residents aged 45 years and older and their spouses (including those aged <45 years) recruited from 150 counties/districts in 28 provinces using multistage, stratified, probability-proportional-to-size sampling to ensure representation of both urban and rural areas. Here, we retrieved baseline data for participants in 2011 and their follow-up data in 2013, 2015, and 2018.

### Data collection

Within the CHARLS, trained interviewers conducted face-to-face surveys using a standardised questionnaire [9]. Baseline data included demographics (age, gender, residence (urban/rural), education (below secondary *vs*. secondary and above), marital status (married *vs*. unmarried/other)), lifestyle factors (smoking, alcohol use, physical activity), and health status (activities of daily living (ADL) score, self-rated health, and number of chronic conditions from a predefined list of 13 diseases, categorised as 0, 1, or ≥2). The CHARLS was approved by Peking University’s Biomedical Ethics Review Committee (IRB 00001052–11015), complied with the Declaration of Helsinki, and obtained written informed consent. As its deidentified data are publicly available, we did not require a separate ethical approval for our study.

### Assessment of CircS

The primary exposure, CircS, was assessed at baseline (2011). Originally proposed by Zimmet and colleagues [5], the diagnostic criteria adopted in this study have been validated and widely applied in Chinese populations [1,3,7,10]. Specifically, CircS comprised seven components across metabolic, physiological, and psychological domains [11–16], with participants meeting ≥4 criteria classified as having CircS [7]:

– central obesity: waist circumference ≥85 cm;

– elevated blood pressure: systolic blood pressure ≥130 mm Hg or diastolic blood pressure ≥85 mm Hg, self-reported physician diagnosis of hypertension, or use of antihypertensive medication;

– low high-density lipoprotein cholesterol: <50 mg/dL;

– elevated triglycerides: ≥150 mg/dL, self-reported physician diagnosis of dyslipidaemia, or use of lipid-lowering medication;

– elevated blood glucose: fasting blood glucose >100 mg/dL, a physician-diagnosed history of diabetes, or use of hypoglycemic medication/insulin;

– abnormal sleep duration: self-reported nightly sleep ≤6 hours or ≥9 hours;

– depressive symptoms: a score of 10 or higher on the 10-item Center for Epidemiologic Studies Depression Scale.

### Ascertainment of outcomes

The primary outcome was incident multimorbidity during the 2013, 2015, and 2018 waves, defined as the first occurrence of a specified pattern in participants free of it at baseline. Health status was assessed across three domains: physical disease, psychological disorder, and cognitive impairment. Physical disease was understood as a self-reported physician diagnosis of hypertension, diabetes, cancer, chronic lung disease, heart disease, stroke, arthritis, or dyslipidaemia. Psychological disorder was classified as the presence of depressive symptoms, indicated by a Center for Epidemiologic Studies Depression Scale score ≥10. Cognitive impairment was defined as a score ≥1.5 standard deviation below the age-specific mean in any domain of immediate or delayed word recall, serial-7 subtraction, or temporal orientation [12,17]. We analysed four specific multimorbidity patterns as outcome: physical-psychological, physical-cognitive, psychological-cognitive, and physical-psychological-cognitive. This framework, based on three health domains, has been established in prior epidemiological studies, demonstrating the prevalence and clinical relevance of these combinations [18,19].

### Statistical analysis

We handled missing covariates *via* multiple imputation (generating five imputed datasets) under a missing-at-random assumption, using mice with all exposure, outcome, and covariates in the imputation model. We summarised baseline characteristics by CircS status and compared them using *t*-tests/analysis of variance for normally distributed continuous variables, Kruskal-Wallis tests for non-normal continuous variables, and χ^2^ tests for categorical variables. We assessed multicollinearity *via* variance inflation factors, with those >5 flagged as concerning.

We visualised the cumulative incidence of each multimorbidity pattern with Kaplan-Meier curves and compared them with log-rank tests. Then, we utilised Cox proportional hazards models to estimate hazard ratios (HRs) and 95% confidence intervals (CIs) across three models: model 1, which was unadjusted; model 2, which was adjusted for age, sex, education, marital status, and residence; model 3, which was further adjusted for smoking, alcohol use, physical activity, ADL score, self-rated health, and baseline number of chronic diseases. We adjusted the *P*-values for the final model for the false discovery rate using the Benjamini-Hochberg procedure. Proportional hazards were checked with Schoenfeld residuals. We examined the dose-response relationship between the number of CircS components and the risk of multimorbidity patterns by modelling the number of CircS components as categorical (0–1 reference, 2–3, ≥4) and continuous (trend). We used restricted cubic splines with three knots to assess nonlinearity.

We further performed subgroup analyses by sex, age (<60 *vs*. ≥60), education, residence, and baseline chronic disease count, testing effect modification *via* multiplicative interaction terms in the fully adjusted model, with Benjamini-Hochberg-adjusted *P*-values for all subgroup and interaction tests. We also analysed a composite endpoint, defined as the first occurrence of any multimorbidity pattern (physical-psychological, physical-cognitive, psychological-cognitive, or physical-psychological-cognitive multimorbidity), and calculated the population attributable fraction (PAF) from the fully adjusted model. Lastly, we performed sensitivity analyses, including a two-year lag analysis that excluded early events; tests of subjective-component influence by excluding participants with baseline depressive symptoms, redefining CircS without depressive symptoms, and redefining CircS using only five objective components (excluding depressive symptoms and sleep duration); a complete-case analysis; and replication using a discrete-time survival model.

We performed all analyses in *R*, version 4.2.2 (R Core Team, Vienna, Austria). All tests were two-sided, with a statistical significance threshold set at *P* < 0.05.

## RESULTS

### Participants’ baseline characteristics

**Figure 1:**
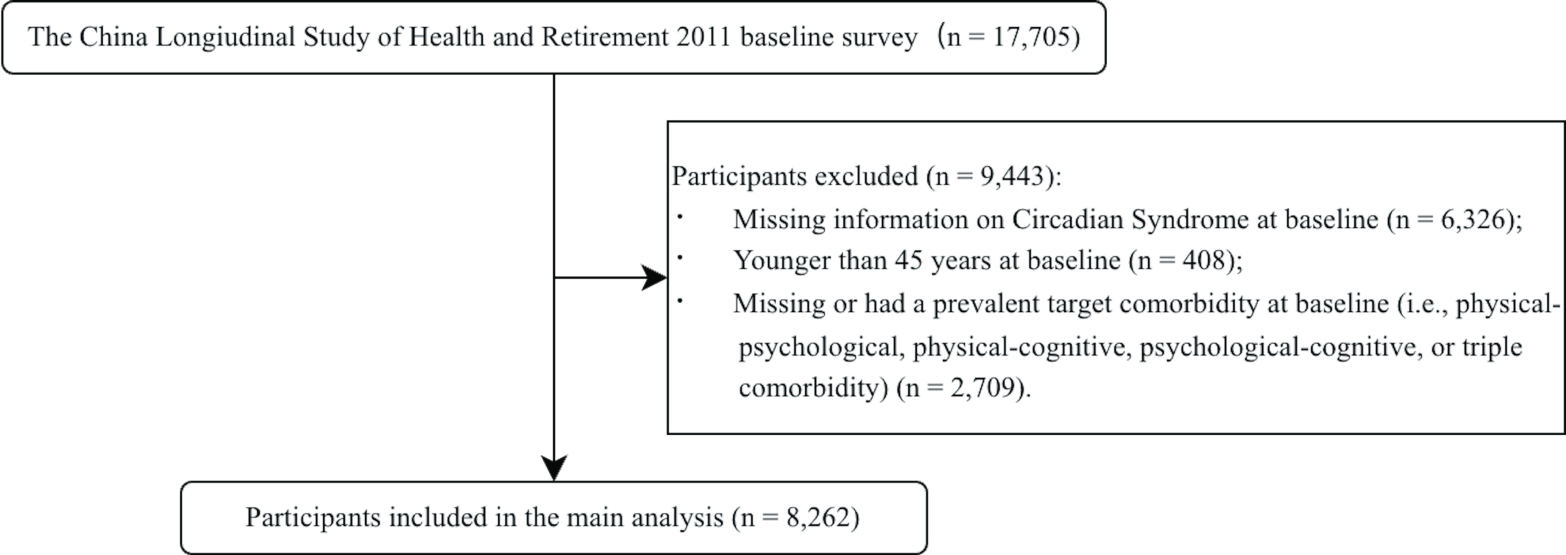
Flowchart of participant selection showing the exclusion criteria applied to the initial cohort from the CHARLS 2011 baseline survey to derive the final analytical sample. CircS – circadian syndrome.

The 2011 baseline included 17 705 participants. We excluded those who were aged <45 years, lacked CircS exposure data, had any specified multimorbidity pattern (physical-psychological, physical-cognitive, psychological-cognitive, or physical-psychological-cognitive), or had missing baseline outcome data. The final cohort comprised 8262 participants, of whom 2925 (35.4%) had CircS at baseline (**Figure 1**, **Table 1**). Compared with individuals without CircS, those with it were older (median age 59 *vs*. 57 years), and had a higher proportion of females (53% *vs*. 49%) and unmarried individuals (14% *vs*. 11%). The CircS group also had a higher proportion of individuals with below-secondary education (89% *vs*. 87%) and residing in urban areas (41% *vs*. 35%). Although participants with CircS reported slightly lower proportions of smoking and alcohol consumption, they had substantially lower levels of physical activity (median of 4158 *vs*. 5544 metabolic equivalent of task). Furthermore, a greater proportion of the CircS group had at least one ADL limitation (17% *vs*. 12%), reported their health as poor (25% *vs*. 18%), and had two or more chronic conditions (41% *vs*. 28%).

**Table 1:** Baseline characteristics of the study participants according to circadian syndrome status*

Characteristic	Overall (n = 8262)	CircS negative (n = 5337)	CircS positive (n = 2925)	*P*-value†
**Age in years, MD (IQR)**	58 (51–65)	57 (51–64)	59 (52–66)	<0.001
**Gender**				<0.001
Female	4137 (50)	2600 (49)	1537 (53)	
Male	4125 (50)	2737 (51)	1388 (47)	
**Education level**				<0.001
Below secondary	7240 (88)	4640 (87)	2600 (89)	
Secondary or above	1022 (12)	697 (13)	325 (11)	
**Marital Status**				<0.001
Married	7288 (88)	4758 (89)	2530 (86)	
Non-married	974 (12)	579 (11)	395 (14)	
**Residence**				<0.001
Rural	5170 (63)	3446 (65)	1724 (59)	
Urban	3092 (37)	1891 (35)	1201 (41)	
**Smoking history**	3360 (41)	2252 (42)	1108 (38)	<0.001
**Drinking history**	3346 (41)	2193 (41)	1153 (39)	<0.001
**MET in MET-hours, MD (IQR)**	5040 (462–12 180)	5544 (560–13 440)	4158 (0–8640)	<0.001
**ADL**				<0.001
0	7040 (86)	4643 (88)	2397 (83)	
1	629 (7.7)	360 (6.8)	269 (9.3)	
2	239 (2.9)	130 (2.5)	109 (3.8)	
3	91 (1.1)	47 (0.9)	44 (1.5)	
4	73 (0.9)	38 (0.7)	35 (1.2)	
5	58 (0.7)	28 (0.5)	30 (1.0)	
6	41 (0.5)	25 (0.5)	16 (0.6)	
**Self-reported health**				<0.001
Excellent	50 (0.8)	33 (0.9)	17 (0.8)	
Very good	560 (9.4)	369 (9.6)	191 (9.1)	
Good	1172 (20)	818 (21)	354 (17)	
Fair	2920 (49)	1915 (50)	1005 (48)	
Poor	1228 (21)	706 (18)	522 (25)	
**Number of chronic diseases**				<0.001
≥2	2609 (33)	1459 (28)	1150 (41)	
0	2991 (38)	2108 (41)	883 (31)	
1	2334 (29)	1561 (30)	773 (28)	

ADL – activities of daily living, CircS – circadian syndrome, MD – median, MET – metabolic equivalent of task, IQR – interquartile range

*Presented as n (%) unless specified otherwise.

†Calculated using the Kruskal-Wallis test for continuous variables and the χ^2^ test for categorical variables.

### Association of CircS with the incidence of four multimorbidity patterns

We detected no significant multicollinearity among the covariates, as all variance inflation factors were <5, while the proportional hazards assumption for our main exposure variable (the CircS score) was tested and met in all Cox models (Tables S1 and S2 in **Online Supplementary Document**).

#### Survival analysis of baseline CircS status and development of multimorbidity

**Figure 2:**
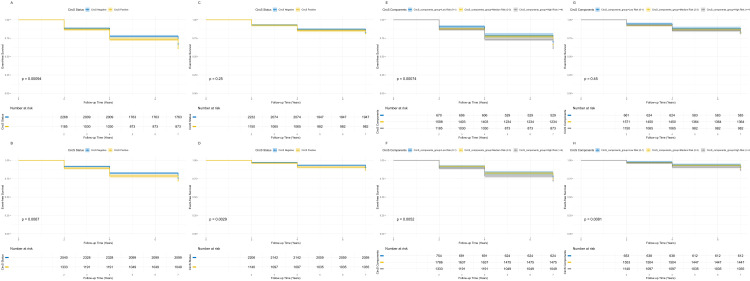
Kaplan-Meier survival estimates and dose-response relationships for multimorbidity patterns according to CircS status. **Panels A–D** show the Kaplan-Meier survival curves for event-free survival probability in participants with CircS (yellow line) versus those without CircS (blue line). **Panel A. **Physical-psychological multimorbidity. **Panel B. **Physical-cognitive multimorbidity. **Panel C.** Psychological-cognitive multimorbidity. **Panel D.** Physical-psychological-cognitive (triple) multimorbidity. Panels E–H show the dose-response relationship based on the number of CircS components (0–1, 2–3, or ≥4). **Panel E.** Physical-psychological multimorbidity. **Panel F.** Physical-cognitive multimorbidity. **Panel G.** Psychological-cognitive multimorbidity. **Panel H.** Physical-psychological-cognitive (triple) multimorbidity. *P*-values were derived from log-rank tests. CircS – circadian syndrome.

All analyses are based on data generated after multiple imputation; missing data proportions were generally <5%, and the fraction of missing information remained <3% (Tables S3 and S4 in **Online Supplementary Document**). The Kaplan-Meier analysis showed that participants with CircS had significantly lower event-free survival for physical-psychological multimorbidity (*P* < 0.001), physical-cognitive multimorbidity (*P* = 0.007), and physical-psychological-cognitive multimorbidity (*P* = 0.003) compared with those without CircS (**Figure 2**). There was no significant association for psychological-cognitive multimorbidity (*P* = 0.25).

We noted a dose-response relationship, where event-free survival for physical-psychological (*P*-value for trend <0.001), physical-cognitive (*P*-value for trend = 0.005), and physical-psychological-cognitive multimorbidity (*P*-value for trend = 0.008) decreased in a stepwise manner with an increasing number of CircS components: 0–1, 2–3, or ≥4. We found no such dose-response trend for psychological–cognitive multimorbidity (*P*-value for trend = 0.45).

#### Prospective association between CircS and multimorbidity risk: Cox regression analysis

After correcting the fully adjusted models for a false discovery rate (**Table 2**), baseline CircS remained significantly associated with an increased risk of triple multimorbidity (HR = 1.48; 95% CI = 1.09, 2.02; *P*  = 0.033) and psychological-cognitive multimorbidity (HR = 1.38; 95% CI = 1.06, 1.79; *P*  = 0.033).The association between CircS and cognitive-physical multimorbidity, however, was no longer statistically significant (HR = 1.22; 95% CI = 1.01, 1.46; *P*  = 0.053). We also found no significant association between CircS and psychological-physical multimorbidity (HR = 1.09; 95% CI = 0.92, 1.30; *P* = 0.301).

**Table 2:** Association between baseline circadian syndrome and the risk of incident multimorbidity patterns*

Outcome	Model	HR (95% CI)	*P*-value	Adjusted *P* -value†
Cognitive-physical multimorbidity	Model 1	1.21 (1.05, 1.39)	0.007	
Cognitive-physical multimorbidity	Model 2	1.26 (1.10, 1.45)	0.001	
Cognitive-physical multimorbidity	Model 3	1.22 (1.01, 1.46)	0.040	0.053
Psychological-cognitive multimorbidity	Model 1	1.04 (0.86, 1.27)	0.692	
Psychological-cognitive multimorbidity	Model 2	1.13 (0.93, 1.38)	0.220	
Psychological-cognitive multimorbidity	Model 3	1.38 (1.06, 1.79)	0.017	0.033
Psychological-physical multimorbidity	Model 1	1.20 (1.05, 1.36)	0.006	
Psychological-physical multimorbidity	Model 2	1.20 (1.05, 1.36)	0.007	
Psychological-physical multimorbidity	Model 3	1.09 (0.92, 1.30)	0.301	0.301
Triple multimorbidity	Model 1	1.29 (1.02, 1.62)	0.033	
Triple multimorbidity	Model 2	1.36 (1.08, 1.72)	0.009	
Triple multimorbidity	Model 3	1.48 (1.09, 2.02)	0.013	0.033

ADL – activities of daily living, CI – confidence interval, HR – hazard ratio, METs – metabolic equivalent of tasks

*Analysis conducted using Cox proportional hazards models based on five imputed data sets. The reference group consists of participants without CircS at baseline. Model 1: crude model, unadjusted. Model 2: adjusted for baseline demographic characteristics, including age, gender, education level, marital status, and residence (urban/rural). Model 3: adjusted for variables in model 2, plus lifestyle and health status variables, including smoking status, drinking status, physical activity (METs), ADLs, and the baseline number of chronic diseases.

**†***P*-values for the four primary outcomes in model 3 were adjusted for multiple comparisons using the Benjamini-Hochberg FDR method.

We further observed a significant dose-response relationship between the number of CircS components and incident multimorbidity in the fully adjusted models (**Table 3**). Compared with participants with 0–1 components, those with ≥4 had a higher risk of developing physical-psychological-cognitive multimorbidity (HR = 1.77; 95% CI = 1.18, 2.65), psychological-cognitive multimorbidity (HR = 1.63; 95% CI = 1.18, 2.25), and physical-cognitive multimorbidity (HR = 1.39; 95% CI = 1.09, 1.77). Similarly, tests for trend were significant for these three outcomes. Each additional CircS component was associated with an increased risk of 37% for physical-psychological–cognitive multimorbidity (HR = per component 1.37; 95% CI = 1.12, 1.67), 27% for psychological-cognitive multimorbidity (HR = 1.27; 95% CI = 1.09, 1.48), and 17% for physical-cognitive multimorbidity (HR = 1.17; 95% CI = 1.04, 1.32). For physical-psychological multimorbidity, the dose-response relationship was not statistically significant (*P*-value for trend = 0.057). The hazard ratio for participants with four or more components *vs*. those with 0–1 was 1.25 (95% CI = 1.00, 1.56; *P* = 0.050).

**Table 3:** Dose-response relationship between the number of circadian syndrome components and the risk of incident multimorbidity patterns*

			

	Model 1		Model 2		Model 3	
**Outcome and CircS component count**	**HR (95% CI)**	***P*-value**	**HR (95% CI)**	***P*-value**	**HR (95% CI)**	***P*-value**
Psychological-physical multimorbidity						
*0–1 components*	ref		ref		ref	
*2–3 components*†	1.17 (1.00, 1.38)	0.056	1.18 (1.01, 1.39)	0.041	1.16 (0.94, 1.43)	0.163
*≥4 components*†	1.36 (1.15, 1.61)	<0.001	1.36 (1.15, 1.61)	<0.001	1.25 (1.00, 1.56)	0.050
*HR per component (trend)*‡	1.17 (1.08, 1.26)	<0.001	1.16 (1.07, 1.26)	<0.001	1.11 (1.00, 1.24)	0.057
Cognitive-physical multimorbidity						
*0–1 components*	ref		ref		ref	
*2–3 components*†	1.18 (0.99, 1.41)	0.070	1.22 (1.02, 1.46)	0.031	1.20 (0.95, 1.52)	0.119
*≥4 components*†	1.35 (1.12, 1.62)	0.002	1.43 (1.19, 1.73)	<0.001	1.39 (1.09, 1.77)	0.009
*HR per component (trend)*‡	1.16 (1.06, 1.26)	0.001	1.19 (1.09, 1.30)	<0.001	1.17 (1.04, 1.32)	0.008
Psychological-cognitive multimorbidity						
*0–1 components*	ref		ref		ref	
*2–3 components*†	1.06 (0.85, 1.33)	0.588	1.14 (0.91, 1.42)	0.255	1.32 (0.97, 1.79)	0.077
*≥4 components*†	1.15 (0.92, 1.45)	0.229	1.30 (1.03, 1.64)	0.029	1.63 (1.18, 2.25)	0.003
*HR per component (trend)* ‡	1.07 (0.96, 1.20)	0.209	1.14 (1.02, 1.28)	0.025	1.27 (1.09, 1.48)	0.003
Triple multimorbidity						
*0–1 components*	ref		ref		ref	
*2–3 components*†	1.15 (0.85, 1.55)	0.364	1.20 (0.89, 1.62)	0.224	1.18 (0.80, 1.74)	0.413
*≥4 components*†	1.51 (1.12, 2.04)	0.007	1.65 (1.22, 2.23)	0.001	1.77 (1.18, 2.65)	0.007
*HR per component (trend)*‡	1.25 (1.08, 1.44)	0.003	1.30 (1.12, 1.51)	<0.001	1.37 (1.12, 1.67)	0.002

ADL – activities of daily living, CI – confidence interval, HR – hazard ratio, METs – metabolic equivalent of tasks, ref – reference

*Analyses conducted using Cox proportional hazards models based on 5 imputed data sets. Model 1: crude model, unadjusted. Model 2: adjusted for baseline demographic characteristics, including age, gender, education level, marital status, and residence (urban/rural). Model 3: adjusted for variables in model 2, plus lifestyle and health status variables, including smoking status, drinking status, physical activity (METs), ADL, and the baseline number of chronic diseases.

†Categorical analysis; reference group consists of participants with 0–1 components of CircS at baseline.

‡Calculated by entering the number of CircS components (treated as a single continuous variable from 0 to 6) into the respective models.

**Figure 3:**
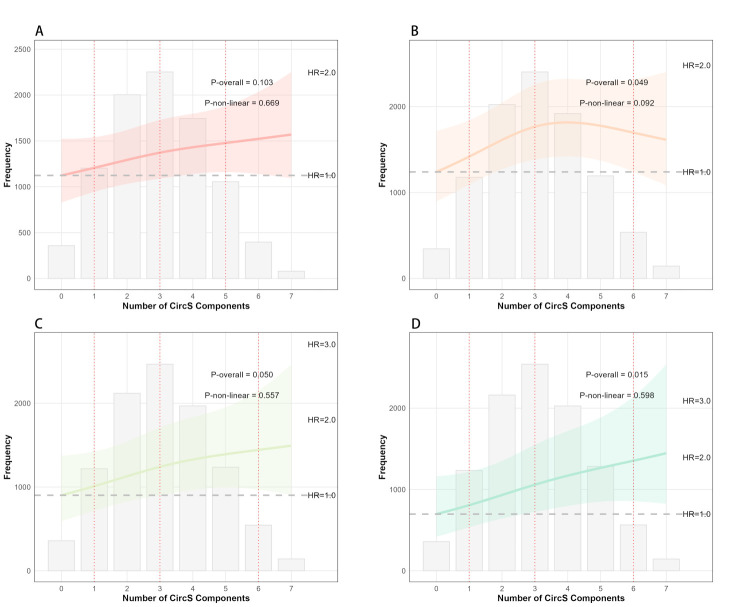
Restricted cubic spline analysis of the association between the number of CircS components and the risk of multimorbidity patterns. The curves illustrate the HRs (solid red/orange/green lines) and 95% CIs (shaded areas) for developing different multimorbidity patterns as the number of CircS components increases. The reference point is set at 0–1 components. **Panel A.** Physical-psychological multimorbidity. **Panel B.** Physical-cognitive multimorbidity. **Panel C.** Psychological-cognitive multimorbidity. **Panel D.** Physical-psychological-cognitive (triple) multimorbidity. CircS – circadian syndrome, HR – hazard ratio, RCS – restricted cubic spline.

The restricted cubic spline analysis (**Figure 3**, Panels A–D) showed a significant linear dose-response relationship between the number of CircS components and the risk of both physical-psychological-cognitive multimorbidity (*P*-value for overall association = 0.015, *P*-value for nonlinearity = 0.60) and psychological-cognitive multimorbidity (*P*-value for overall association = 0.050, *P*-value for nonlinearity = 0.56). There was also a significant overall association for physical-cognitive multimorbidity (*P*-value for overall association = 0.049), with some evidence of a nonlinear relationship (*P*-value for nonlinearity = 0.092). For physical-psychological multimorbidity, the overall association was not statistically significant (*P*-value for overall association = 0.10).

#### Heterogeneity of risk association: subgroup analysis and effect modification

After applying a Benjamini-Hochberg correction, we found no statistically significant effect modification by sex, age, educational attainment, residence, or baseline number of chronic conditions (Table S5 in the **Online Supplementary Document**). However, stratified analyses revealed that the associations between CircS and multimorbidity remained statistically significant in several key subgroups after correcting for a false discovery rate. Notably, the risk associated with CircS was consistently elevated among women across all multimorbidity patterns. We also noted significant associations in participants with lower educational attainment and those with no chronic conditions at baseline.

#### Comprehensive risk assessment and public health significance

The association between CircS and the composite outcome was significant across all levels of adjustment (Table S6 in the **Online Supplementary Document**). The HRs were 1.16 (95% CI = 1.05, 1.27) in the crude model, 1.18 (95% CI = 1.07, 1.30) after adjustment for sociodemographic factors, and 1.14 (95% CI = 1.00, 1.29; *P* = 0.047) in the final, fully adjusted model.

The PAF was highest for physical-psychological-cognitive multimorbidity, with an estimated 16.8% (95% CI = 6.6–27.2) of cases statistically attributable to CircS (Table S7 in the **Online Supplementary Document**). It was also significant for psychological-cognitive multimorbidity (10.7%; 95% CI = 2.5–19.2) and physical-cognitive multimorbidity (7.0%; 95% CI = 0.9, 13.3), but not for physical-psychological multimorbidity (4.1%; 95% CI = –1.4, 9.7).

The findings were robust in several sensitivity analyses (Tables S8–14 in the **Online Supplementary Document**). First, the primary associations remained significant after excluding participants with baseline depressive symptoms (CES-D score ≥10) and in a two-year lag analysis that excluded events occurring within the first two years of follow-up to account for potential reverse causality. The main associations also remained largely consistent after we redefined CircS using six components (*i.e.*, after excluding the depressive symptom component). A complete case analysis yielded largely consistent results, with the magnitude of associations being maintained or strengthened for most outcomes. Fifth, when redefining CircS to include only five objective components (excluding depressive symptoms and sleep duration), the positive associations with incident cognitive-physical multimorbidity persisted, whereas associations with outcomes involving psychological conditions were attenuated to non-significance. Finally, the replicated analysis that used a discrete-time survival model yielded highly consistent results, confirming the robustness of our findings to the proportional hazards assumption.

## DISCUSSION

This large, prospective cohort of middle-aged and older Chinese adults provides the first longitudinal evidence that CircS is a significant predictor of incident multimorbidity, particularly for complex patterns involving cognitive and psychological decline. Specifically, we found that individuals with CircS had a 48% higher risk of developing physical-psychological-cognitive (triple) multimorbidity and a 38% higher risk of developing psychological-cognitive multimorbidity. We also observed a clear dose-response relationship, where each additional CircS component incrementally increased the risk for these outcomes. These findings underscore CircS as a critical, integrative risk factor that precedes the development of complex disease clusters in aging populations.

Our results extend previous research which has largely focussed on the association between individual circadian-related factors (*e.g.* sleep duration, metabolic syndrome) and single diseases [6–8]. While studies have linked metabolic syndrome to cognitive decline [20] and sleep disruption to depression [21], our study is the first to conceptualise these factors within the unified CircS framework and prospectively link it to the onset of multimorbidity patterns. The strong association with triple multimorbidity is particularly novel and clinically relevant: it suggests that the co-occurrence of metabolic, sleep, and mood dysregulation – the core of CircS – may trigger a synergistic cascade of pathology that simultaneously impacts physical, cognitive, and mental health domains, rather than these domains deteriorating independently. This aligns with emerging evidence that highlights shared pathophysiological pathways, such as systemic inflammation and neuroendocrine dysregulation, as common drivers of diverse chronic conditions [22].

Several plausible biological mechanisms may explain the observed associations. First, chronic circadian disruption, central to CircS, promotes a state of low-grade systemic inflammation and oxidative stress through the dysregulation of clock genes that govern immune and metabolic pathways [23]. This pro-inflammatory state is a well-established risk factor for both neurodegenerative processes (contributing to cognitive impairment) and cardiometabolic diseases (contributing to physical conditions). Second, sleep disruption, a core component of CircS, impairs the glymphatic system’s clearance of neurotoxic waste products like amyloid-beta, directly increasing the risk for cognitive decline [24], while also exacerbating insulin resistance and hypertension, linking it to physical disease [10]. Third, the psychological component of CircS, depressive symptoms, is linked to hyperactivity of the hypothalamic-pituitary-adrenal axis and elevated cortisol levels, which can induce neuronal damage in the hippocampus and prefrontal cortex, thereby connecting mood disorders with cognitive deficits [25]. CircS, therefore, represents a convergence of these interconnected pathways, making it a powerful upstream driver of multimorbidity.

Importantly, we found that the association between CircS and multimorbidity was consistently stronger in women and in individuals who were free of chronic conditions at baseline. This may be attributable to sex differences in hormonal profiles, sleep architecture, and the prevalence of depression, which could amplify the adverse effects of circadian disruption [10,25,26]. Our observation of a stronger association in initially healthy individuals is particularly compelling; we hypothesise that CircS may act as a potent initiator of the multimorbidity cascade, with its effects being most pronounced before the confounding influence of pre-existing diseases obscures the signal. This highlights the importance of targeting CircS as a primary prevention strategy in healthy aging populations.

Our findings have significant clinical and public health implications. The observed dose-response relationship provides a clear basis for risk stratification and suggests that CircS could be a valuable screening tool for identifying individuals at high risk of multimorbidity, reinforcing the need for integrated management models targeting metabolic, sleep, and psychological health [1,26]. At the public health level, our estimate that nearly 17% of the most complex multimorbidity cases were statistically attributable to CircS highlights the potential public health relevance of this association and points to a substantial area for preventive medicine research. While this finding does not imply causality, it suggests that interventions promoting circadian health – such as encouraging regular sleep-wake cycles, optimising meal timing, and ensuring adequate daylight exposure – could help mitigate the rising prevalence of multimorbidity, and should thus be tested in further studies [28]. Moreover, effective public health strategies must also address the societal drivers of circadian disruption, such as shift work, urbanisation, and light pollution [10,26,28]. This calls for complementary occupational and environmental policies that create healthier living and working conditions.

Our study has several strengths. Its prospective design, based on a large, nationally representative cohort, allowed us to establish temporal relationships and minimise recall bias by assessing exposure prior to outcome onset and incorporating objective biomarkers. Our systematic examination of four distinct multimorbidity patterns provides a nuanced understanding beyond a simple count of diseases. Furthermore, we confirmed the robustness of our findings through several sensitivity analyses, including a lag analysis to account for reverse causality, redefinitions of the CircS score to test the influence of subjective components, and the use of a discrete-time survival model to validate the proportional hazards assumption.

This study also has several limitations. First, while metabolic components were objectively measured, the assessment of sleep duration and depressive symptom relied on self-reported data, without objective validation from biomarkers like actigraphy. As such, the score should be considered a proxy measure, potentially influenced by recall or reporting bias. Consequently, our observational design identifies a significant longitudinal association, rather than an independent causal risk factor, and residual confounding cannot be excluded. Second, our multimorbidity outcome was also based on a count of self-reported diseases, introducing a risk of reporting bias and common method variance, particularly as depressive symptoms might influence the reporting of physical illnesses. While the above-mentioned sensitivity analyses indicated that the main associations remained robust, this issue of conceptual overlap was not entirely resolved. Third, our analysis did not formally account for the competing risk of mortality using methods like the Fine-Gray model. The reason for this was the limited reliability of the cause-of-death data in the CHARLS survey, which can be incomplete or lack the systematic adjudication necessary for accurate cause-specific hazard modelling. Fourth, all subgroup analyses were exploratory only and intended for hypothesis generation; thus, these findings should be interpreted with caution. Finally, the findings from this Chinese cohort require external validation in other populations to establish generalisability.

## CONCLUSIONS

This large prospective study provides the first longitudinal evidence that CircS is a significant risk factor for the development of complex multimorbidity, particularly patterns involving cognitive and psychological decline. The observed dose-response relationship suggests that CircS acts as an integrative stressor, where metabolic, sleep, and mood dysregulation synergistically accelerate the onset of concurrent physical, psychological, and cognitive conditions. These findings establish the condition as a critical target for both clinical risk stratification and multifaceted public health strategies. Such strategies must not only promote individual circadian health, but also address the broader societal drivers of circadian disruption to effectively mitigate the growing burden of multimorbidity.

## Additional material


Online Supplementary Document
