# Supplementary material for: Association of circadian syndrome with the risk of physical, psychological, and cognitive multimorbidities: a prospective cohort study based on the China Health and Retirement Longitudinal Study
Source: J Glob Health. 2025 Dec 19;15:04351. doi: 10.7189/jogh.15.04351 (PMC12714357; doi:10.7189/jogh.15.04351)
Supplement: Online Supplementary Document [file jogh-15-04351-s001.pdf]

Supplement to: Bi J, Pan Y, Guo W, Ji P, Xing Z, Feng L, Xie Y. Association of circadian syndrome with the risk of physical, psychological, and cognitive multimorbidities: a prospective cohort study based on the China Health and Retirement Longitudinal Study. J Glob Health. 2025;15:04351.

Supplementary Table 1. Multicollinearity Diagnostics for Covariates Included in the Cox Proportional Hazards Models.

Variable	VIF
Gender	1.498998
Education	1.041625
Marital	1.015328
Residence	1.045557
Smoking	1.386727
Drinking	1.231092
MET	1.024500
ADL	1.050100
Health	1.024197
Chronic_disease_count	1.032905

Notes: VIF, Variance Inflation Factor. A VIF value < 5 is generally considered to indicate the absence of serious multicollinearity among the variables. *Abbreviations:* MET, Metabolic Equivalent of Task; ADL, Activities of Daily Living.

Supplementary Table 2. Test of the Proportional Hazards Assumption for the Association of the CircS Score with Incident Multimorbidity Patterns.

Outcome Domain	p-value for the CircS Score	Proportional Hazards Assumption
Psychological-Physical Multimorbidity	0.507	Met
Cognitive-Physical Multimorbidity	0.087	Met
Psychological-Cognitive Multimorbidity	0.620	Met
Triple Multimorbidity	0.243	Met

Notes: The proportional hazards assumption was evaluated based on Schoenfeld residuals from the fully adjusted Cox proportional hazards models. A p-value > 0.05 indicates that the assumption is not violated for the variable.

Supplementary Table 3. Fraction of Missing Information (FMI) for the Primary Exposure Variable After Multiple Imputation

Outcome Model	Fraction of Missing Information (FMI)
Physical-Psychological Multimorbidity	2.9%
Physical-Cognitive Multimorbidity	1.8%
Psychological-Cognitive Multimorbidity	2.0%
Physical-Psychological-Cognitive Multimorbidity	1.1%

Notes: The Fraction of Missing Information (FMI) quantifies the proportion of variance in the final estimate that is attributable to the missing data. The consistently low FMI values (<3%) across all four outcome models suggest that the missing data had a minimal impact on the precision of the association estimates for the primary exposure variable (CircS_diagnosis). Results are based on pooling five imputed datasets.***Abbreviations:*** FMI, Fraction of Missing Information.

Supplementary Table 4. Proportion of Missing Data for Covariates Prior to Multiple Imputation

Variable	Missing Count	Missing Proportion (%)
Age	0	0.0
Gender	0	0.0
Education	0	0.0

Marital	0	0.0
Residence	0	0.0
Smoking	2	0.02
Drinking	9	0.11
MET	4768	57.7
ADL	91	1.1
Health	2332	28.2
Chronic_disease_count	328	4.0

Notes: The table displays the absolute count and corresponding percentage of missing values for each covariate included in the analytical models. Percentages are based on the total analysis sample (N = 8,261). These variables were subsequently included in the multiple imputation procedure.***Abbreviations*:** ADL, Activities of Daily Living; MET, Metabolic Equivalent of Task.

Supplementary Table 5. Subgroup and Interaction Analysis of the Association Between Circadian Syndrome (CircS) and Incident Multimorbidity Patterns.

**Characteristic**	**Subgroup**	**HR (95% CI)**	**P-value (Subgroup)**	**P-adj (Subgroup)**	**P for Interaction**	**P-adj (Interaction)**
**Physical–Psychological Multimorbidity**						
**Sex**		-	-	-	0.444	0.888
	Female	1.33 (1.07–1.65)	0.010	0.038		
	Male	1.12 (0.91–1.38)	0.282	0.355		
**Age**		-	-	-	0.644	0.984
	<60 years	1.20 (0.99–1.47)	0.070	0.140		
	⩾60 years	1.20 (0.95 – 1.51)	0.133	0.210		
**Education**		-	-	-	0.983	0.984
	< Lower secondary	1.19 (1.02–1.40)	0.030	0.078		
	Secondary or above	1.09 (0.70–1.70)	0.704	0.755		
**Residence**		-	-	-	0.703	0.984
	Urban	1.18 (0.92–1.51)	0.200	0.278		
	Rural	1.21 (1.00–1.46)	0.048	0.106		
**Baseline Chronic Conditions***		-	-	-	0.003	0.073
	0	1.43 (1.08–1.89)	0.012	0.042		
	1	1.23 (0.94–1.62)	0.132	0.210		
	⩾2 (Ref)	0.90 (0.70 – 1.17)	0.440	0.524		
**Physical–Cognitive Multimorbidity**						
**Sex**		-	-	-	0.057	0.229
	Female	1.46 (1.15–1.84)	0.002	0.016		
	Male	1.05 (0.83–1.34)	0.676	0.744		
**Age**		-	-	-	0.912	0.984
	<60 years	1.25 (1.01–1.55)	0.039	0.094		
	⩾60 years	1.25 (0.94 – 1.65)	0.120	0.210		
**Education**		-	-	-	0.028	0.204
	< Lower secondary	1.33 (1.12–1.58)	0.001	0.016		
	Secondary or above	0.57 (0.29–1.12)	0.112	0.205		
**Residence**		-	-	-	0.139	0.418
	Urban	1.49 (1.11–1.99)	0.009	0.038		
	Rural	1.15 (0.94–1.42)	0.175	0.256		
**Baseline Chronic Conditions***		-	-	-	0.256	0.615
	0	1.50 (1.11–2.03)	0.010	0.038		
	1	1.08 (0.80–1.45)	0.620	0.718		
	⩾2 (Ref)	1.17 (0.87 – 1.56)	0.298	0.364		

**Psychological–Cognitive Multimorbidity**						
**Sex**		-	-	-	0.053	0.229
	Female	1.65 (1.24–2.20)	<0.001	0.012		
	Male	1.01 (0.70–1.44)	0.976	0.976		
**Age**		-	-	-	0.369	0.804
	<60 years	1.24 (0.94–1.62)	0.125	0.210		
	⩾60 years	1.64 (1.11 - 2.42)	0.015	0.047		
**Education**		-	-	-	0.939	0.984
	< Lower secondary	1.36 (1.08–1.71)	0.009	0.038		
	Secondary or above	1.15 (0.44–2.96)	0.787	0.816		
**Residence**		-	-	-	0.673	0.984
	Urban	1.25 (0.84–1.85)	0.274	0.354		
	Rural	1.38 (1.06–1.80)	0.019	0.056		
**Baseline Chronic Conditions***		-	-	-	0.543	0.984
	0	1.33 (0.95–1.86)	0.093	0.179		
	1	1.36 (0.88–2.11)	0.170	0.256		
	⩾2 (Ref)	1.34 (0.86 - 2.09)	0.202	0.278		
**Physical–Psychological–Cognitive Multimorbidity**						
**Sex**		-	-	-	0.034	0.204
	Female	2.13 (1.50–3.01)	<0.001	0.002		
	Male	1.10 (0.71–1.71)	0.676	0.744		
**Age**		-	-	-	0.856	0.984
	<60 years	1.61 (1.15–2.25)	0.006	0.035		
	⩾60 years	1.70 (1.07 - 2.69)	0.029	0.078		
**Education**		-	-	-	0.980	0.984
	< Lower secondary	1.62 (1.23–2.14)	<0.001	0.012		
	Secondary or above	1.31 (0.36–4.77)	0.797	0.816		
**Residence**		-	-	-	0.984	0.984
	Urban	1.63 (1.02–2.60)	0.046	0.106		
	Rural	1.60 (1.15–2.23)	0.006	0.035		
**Baseline Chronic Conditions***		-	-	-	0.123	0.418
	0	2.05 (1.25–3.35)	0.006	0.035		
	1	1.64 (1.00–2.70)	0.055	0.116		
	⩾2 (Ref)	1.33 (0.85 - 2.09)	0.222	0.296		

Notes:P-adj (Subgroup) and P-adj (Interaction) values were calculated separately using the Benjamini-Hochberg procedure. P-adj (Subgroup) corrects for all 44 subgroup tests. P-adj (Interaction) corrects for all 24 interaction tests.*For Baseline Chronic Conditions, which has more than two levels, the lowest P-value among the interaction terms is reported.***Abbreviations*****:**HR, Hazard Ratio; CI, Confidence Interval; P-adj, P-value adjusted for False Discovery Rate (FDR); Ref, Reference group.

Supplementary Table 6. Association Between Circadian Syndrome and the Risk of the Composite Multimorbidity Endpoint

Model	HR (95% CI)	P-value
**Model 1**	1.16 (1.05–1.27)	0.004
**Model 2**	1.18 (1.07–1.30)	0.001
**Model 3**	1.14 (1.00–1.29)	**0.047**

Notes**:** Hazard ratios (HRs) and 95% CIs were estimated from Cox proportional hazards models using multiply imputed data. The analysis was restricted to participants free of any multimorbidity pattern at baseline. The

composite outcome was the first incidence of physical–psychological, physical–cognitive, psychological–cognitive, or physical–psychological–cognitive multimorbidity. CI=confidence interval.

Supplementary Table 7. Population Attributable Fraction for the Association of Circadian Syndrome with the Incidence of Different Multimorbidity Patterns

Outcome	Prevalence of CircS	Adjusted HR	Population Attributable Fraction (PAF, 95% CI)
**Psychological-Physical Multimorbidity**	35.40%	1.12	4.08% (-1.36% to 9.71%)
**Cognitive-Physical Multimorbidity**	35.40%	1.21	6.99% (0.85% to 13.34%)
**Psycho-Cognitive Multimorbidity**	35.40%	1.34	10.70% (2.53% to 19.15%)
**Triple Multimorbidity**	35.40%	1.57	16.77% (6.57% to 27.18%)

Notes: PAFs were calculated to estimate the proportion of incident cases attributable to CircS. Estimates are based on hazard ratios from fully adjusted Cox models that included age, sex, educational attainment, marital status, residence, smoking, alcohol consumption, physical activity, Activities of Daily Living (ADL) score, and baseline number of chronic diseases. For each multimorbidity pattern, the prevalence and hazard ratio were specific to the cohort of participants free of that pattern at baseline. Results from multiply imputed datasets were pooled. ADL=Activities of Daily Living. CircS=Circadian Syndrome. PAF=population attributable fraction.

Supplementary Table 8. Association between Circadian Syndrome (CircS) and Incident Multimorbidity in a Sensitivity Analysis Excluding Participants with Baseline Depression

Outcome	Model 1 HR (95% CI)	Model 2 HR (95% CI)	Model 3 HR (95% CI)
**Psychological-Physical Multimorbidity**	1.20 (1.05-1.36)	1.20 (1.05-1.36)	1.09 (0.92-1.30)
**P-value**	0.006	0.007	0.301
**Cognitive-Physical Multimorbidity**	1.21 (1.05-1.39)	1.26 (1.10-1.45)	1.22 (1.01-1.46)
**P-value**	0.007	0.001	0.040
**Psychological-Cognitive Multimorbidity**	1.04 (0.86-1.27)	1.13 (0.93-1.38)	1.38 (1.06-1.79)
**P-value**	0.692	0.220	0.017
**Triple Multimorbidity**	1.29 (1.02-1.62)	1.36 (1.08-1.72)	1.48 (1.09-2.02)
**P-value**	0.033	0.009	0.013

Notes:This sensitivity analysis excluded all participants who had depression (defined by CES-D-10 score ≥ 10) at the baseline survey.HR: Hazard Ratio; CI: Confidence Interval.Model 1: Unadjusted.Model 2: Adjusted for Age, Gender, Education, Marital status, and Residence.Model 3: Adjusted for Model 2 variables plus Smoking status, Drinking status, Physical activity (MET), Activities of Daily Living (ADL), Self-rated health, and the baseline count of chronic diseases.

Supplementary Table 9. Dose-Response Relationship between the Number of CircS Components and Incident Multimorbidity in a Sensitivity Analysis Excluding Participants with Baseline Depression

Outcome and CircS Components	Model 1 HR (95% CI)	Model 2 HR (95% CI)	Model 3 HR (95% CI)
**Psychological-Physical Multimorbidity**			
**0-1 components**	Ref	Ref	Ref
**2-3 components**	1.19 (1.00-1.41)	1.22 (1.03-1.44)	1.13 (0.91-1.41)

**⩾4 components**	1.35 (1.13-1.61)	1.37 (1.15-1.64)	1.20 (0.95-1.52)
**P for trend**	<0.001	<0.001	0.145
**Cognitive-Physical Multimorbidity**			
**0-1 components**	Ref	Ref	Ref
**2-3 components**	1.21 (1.00-1.45)	1.26 (1.05-1.52)	1.24 (0.97-1.58)
**⩾4 components**	1.38 (1.14-1.67)	1.49 (1.22-1.81)	1.42 (1.10-1.83)
**P for trend**	0.001	<0.001	0.008
**Psychological-Cognitive Multimorbidity**			
**0-1 components**	Ref	Ref	Ref
**2-3 components**	0.93 (0.73-1.18)	1.02 (0.80-1.29)	1.09 (0.79-1.52)
**⩾4 components**	0.99 (0.77-1.28)	1.14 (0.89-1.48)	1.47 (1.04-2.07)
**P for trend**	0.971	0.270	0.022
**Triple Multimorbidity**			
**0-1 components**	Ref	Ref	Ref
**2-3 components**	1.17 (0.86-1.59)	1.25 (0.91-1.71)	1.18 (0.78-1.77)
**⩾4 components**	1.43 (1.04-1.97)	1.59 (1.15-2.20)	1.66 (1.08-2.55)
**P for trend**	0.021	0.004	0.013

Notes:This sensitivity analysis excluded all participants who had depression (defined by CES-D-10 score ≥ 10) at the baseline survey.HR: Hazard Ratio; CI: Confidence Interval.Model 1: Unadjusted.**Model 2 (DemographicModel 2: Adjusted for Age, Gender, Education, Marital status, and Residence.Model 3 : Adjusted for Model 2 variables plus Smoking status, Drinking status, Physical activity (MET), Activities of Daily Living (ADL), Self-rated health, and the baseline count of chronic diseases.*P for trend* was calculated by modeling the CircS component categories (0-1, 2-3, ≥4) as a continuous variable (coded 1, 2, 3) in the respective models.

Supplementary Table 10. Sensitivity Analysis: Association between Circadian Syndrome and Multimorbidity Risk using a 2-Year Lagged Analysis.

Outcome	HR (95% CI)	P-value
Psycho-somatic multimorbidity	1.18 (0.97-1.42)	0.093
Cognitive-somatic multimorbidity	1.15 (0.92-1.43)	0.216
Psycho-cognitive multimorbidity	1.37 (1.02-1.84)	0.036
Triple multimorbidity	1.48 (1.05-2.09)	0.027

Notes:The analysis was performed by excluding all incident cases of multimorbidity that occurred within the first two years of follow-up. Hazard Ratios (HRs) and 95% Confidence Intervals (CIs) were derived from fully adjusted Cox proportional hazards models. Models were adjusted for age, gender, education, marital status, residence, smoking, drinking, physical activity (METs), activities of daily living (ADL), self-rated health, and the baseline count of chronic diseases.

Supplementary Table 11. Sensitivity Analysis: Associations of a 6-Component CircS (Excluding Depressive Symptoms) as a Continuous Variable with Incident Multimorbidity Patterns.

Outcome	HR (95% CI)	P-value
Psychological-Physical Multimorbidity	1.02 (0.97-1.07)	0.388
Cognitive-Physical Multimorbidity	1.07 (1.01-1.13)	0.023
Psychological-Cognitive Multimorbidity	1.06 (0.99-1.14)	0.108
Triple Multimorbidity	1.13 (1.03-1.24)	0.009

Notes: The CircS score was treated as a continuous variable ranging from 0 to 6, with hazard ratios representing the risk per one-component increase. Cox proportional hazards models were adjusted for age, gender, education, marital status, residence, smoking status, drinking status, metabolic equivalent tasks (METs), activities of daily living (ADL), self-rated health, and the number of chronic diseases.***Abbreviations:*** CircS, Circulation-Sensation System; HR, Hazard Ratio; CI, Confidence Interval.

Supplementary Table 12. Sensitivity Analysis: Association between CircS and Incident Multimorbidity using Complete Case Analysis

Outcome	N (Events)	HR (95% CI)	P-value
Psychological-Physical Multimorbidity	1016 (346)	1.23 (0.98-1.54)	0.069
Cognitive-Physical Multimorbidity	1128 (288)	1.21 (0.95-1.54)	0.122
Psychological-Cognitive Multimorbidity	997 (156)	1.68 (1.22-2.33)	0.002
Triple Multimorbidity	982 (88)	2.22 (1.44-3.42)	<0.001

Notes: The complete case analysis was restricted to 2,406 participants who had no missing data for all covariates included in the fully adjusted model. Hazard ratios were derived from Cox proportional hazards models adjusted for age, gender, education, marital status, residence, smoking, drinking, physical activity (METs), activities of daily living (ADL), self-rated health, and baseline chronic disease count.***Abbreviations:*** HR, hazard ratio; CI, confidence interval.

Supplementary Table 13. Sensitivity Analysis: Associations of a 5-component CircS Score (Objective Indicators Only) with Incident Multimorbidity

Outcome	HR (95% CI) per 1-point increase	P-value
Psychological-Physical Multimorbidity	0.99 (0.94-1.05)	0.839
Cognitive-Physical Multimorbidity	1.07 (1.01-1.13)	0.022
Psychological-Cognitive Multimorbidity	1.01 (0.93-1.09)	0.851
Triple Multimorbidity	1.09 (0.99-1.20)	0.078

Notes:The 5-component CircS score was calculated as a continuous variable (ranging from 0 to 5) based on five objective indicators: central obesity, elevated blood pressure, reduced HDL-C, elevated triglycerides, and elevated glucose. Self-reported indicat2ors (depressive symptoms and short/long sleep duration) were excluded.Models were adjusted for age, gender, education, marital status, residence, smoking, drinking, physical activity (METs), activities of daily living (ADL) limitations, self-rated health, and the number of chronic diseases at baseline.***Abbreviations:***CircS, Circulatory Health Syndrome; CI, Confidence Interval; HR, Hazard Ratio; HDL-C, High-density lipoprotein cholesterol; MET, Metabolic equivalent.

Supplementary Table 14. Associations of Circulatory Health Score (CircS) with Incident Multimorbidity using Discrete-Time Survival Models

Outcome	OR (95% CI)	P-value
Psychological-Physical Multimorbidity	1.14 (0.97-1.35)	0.117
Cognitive-Physical Multimorbidity	1.24 (1.03-1.49)	0.020
Psychological-Cognitive Multimorbidity	1.38 (1.09-1.74)	0.007
Triple Multimorbidity	1.62 (1.23-2.15)	<0.001

Notes: The discrete-time survival model was implemented using logistic regression on a person-period dataset,

with the time interval (period) included as a factor. The model was fully adjusted for age, gender, education, marital status, residence, smoking status, drinking status, physical activity (METs), activities of daily living (ADL) dependency, self-rated health, and the number of chronic diseases at baseline. Odds Ratios (ORs) from discrete-time models can be interpreted as an approximation of Hazard Ratios (HRs) for the risk of an event in any given time interval, particularly when the event rate is low.***Abbreviations:*** CircS, Circulatory Health Score; CI, confidence interval; OR, odds ratio; MET, metabolic equivalent of task.
